# A predictive model of non-suicidal self-injury - a study based on the construction and validation of a nomogram

**DOI:** 10.3389/fpsyt.2025.1539884

**Published:** 2025-04-04

**Authors:** YuJie Liu, TaiMin Wu, Shu Yan, Yang Zhou, Lianzhong Liu

**Affiliations:** ^1^ School of Medicine, Jianghan University, Wuhan, Hubei, China; ^2^ Office of Psychosocial Services, Wuhan Mental Health Center, Wuhan, Hubei, China; ^3^ Department of Psychiatry, Wudong Hospital, Wuhan, Hubei, China

**Keywords:** machine learning, neural networks, data analysis, bioinformatics, clinical applications

## Abstract

**Background:**

The issue of psychological maladjustment, particularly Non-Suicidal Self-Injury (NSSI), is prevalent among vocational high school students. Therefore, timely identification of high-risk individuals is important in providing further intervention.

**Methods:**

A survey was conducted among 2081 students from a vocational high school in Wuhan, China. The students were divided into two groups: those who had engaged in Non-Suicidal Self-Injury (NSSI) within the past two weeks and those who had not. Lasso regression and logistic regression were employed to identify significant risk factors associated with NSSI. Subsequently, a nomogram was developed to enhance the accuracy and efficiency of identifying individuals at high risk for NSSI. The performance of the model was assessed through various validation methods including Area Under the Curve (AUC), calibration curves, and Decision Curve Analysis (DCA).

**Results:**

The significant predictors of NSSI encompassed gender, problem behavior, depressive mood, and borderline personality tendencies. Based on these predictors, a nomogram was constructed. The model’s accuracy was validated using AUC, calibration curves, and DCA, showing high accuracy.

**Conclusion:**

A nomogram prediction tool for NSSI among vocational high school students was constructed, providing an accurate and quick method for predicting adolescent NSSI behavior.

## 1 Introduction

Non-Suicidal Self-Injury (NSSI) is an increasingly serious clinical and public health problem (1, 2), characterized by individuals repeatedly inflicting superficial but painful physical injuries without suicidal intent. The purpose is mostly to relieve negative emotions or reduce interpersonal stress (3). The onset of NSSI is usually in early adolescence (12 - 14 years). Recent studies on Chinese adolescents have reported a six-month prevalence rate of 22.7% for NSSI (4); Globally, studies estimate a 17% prevalence of non-suicidal self-injury (NSSI) among adolescents, with rates dropping to 6% in adults (5, pp. 1990–2015); this suggests that the lifetime prevalence of this behavior decreases with age. However, failure to intervene promptly can also lead to serious consequences such as cognitive impairment, poor interpersonal relationships, violent crime, and even suicide (6–10). Studies have shown that nearly half (49.29%) of the respondents who had engaged in NSSI behaviors had a history of suicide attempts (11, 12).

There are more risk factors associated with NSSI, which can be broadly categorized into the following seven groups: psychiatric disorders (depression, personality disorders, etc.), bullying, low mental health literacy, problematic behaviors (addictions, substance abuse, etc.), adverse childhood experiences, physical symptoms (disabilities, etc.), and females (13–17). Of these, depression and Borderline Personality Disorder (BPD) are strong correlates of NSSI (18, 19). Problematic behaviors such as addictions, substance abuse, and other behaviors are similarly high-risk factors for NSSI (20, 21).

Vocational education plays an important role in China’s education system, but social recognition of vocational education needs to be improved. Some of the students receiving vocational education may have some challenges and disturbances in terms of academic performance, discipline, character, and habits, which may also affect their self-perception, motivation, and social performance. In addition to the psychological conflicts that characterize adolescence, secondary students also have to face social opinion and employment pressure, which makes them more vulnerable to physical and mental health crises (22, 23). Some studies have shown that the detection rate of NSSI among Chinese secondary school students ranges from 28.9% to 57% (24), which is higher than that of general secondary school students (17%) (25).

Current research on NSSI prediction exhibits two critical limitations. First, while nomograms are widely utilized in clinical depression screening, no dedicated models exist for vocational education populations—particularly secondary vocational students who face unique career-planning pressures and social identity challenges. Second, traditional logistic regression methods often suffer from prediction bias due to multicollinearity in psychological scale data. This study introduces a novel approach by integrating Lasso regression with logistic regression. The methodology involves two phases: (1) Lasso regression automatically identifies key predictors, eliminating redundant variables; (2) logistic regression quantifies risk contributions to generate a visual nomogram. Enable schools, community agencies, and healthcare professionals to swiftly identify individuals at high risk of NSSI.

## 2 Methods

### 2.1 Study population

This large-scale cohort study enrolled all full-time students (N=2,160) aged 16-18 years from a vocational high school in Wuhan during September 2022. The inclusion criteria required: Complete responses on core psychosocial measures (NSSI, depressive symptoms, etc.); Guardian-approved informed consent. Exclusion criteria eliminated: Questionnaires with >20% missing items in key domains (n=51); Invalid responses showing response bias (e.g., identical scores across 90% items, n=28); Participants outside the 16-18 age range (n=0). The final analytical sample comprised 2,081 students (916 males, 1,165 females), representing 96.34% of initial participants. The study was approved by the Ethics Committee of Wuhan Mental Health Center (Ethics No. KY2021.11.01), and informed consent was obtained from the participating students and their guardians.

### 2.2 Measures

Demographic characteristics, school bullying, critical incidents, emotional problems (depression and anxiety), problem behaviors (smoking, drinking, gambling, substance abuse behaviors), and borderline personality disorder (BPD) were investigated.

Demographics: the subjects’ gender, grade level, whether they were an only child, parent’s education level and occupation, and family economic status were mainly included.

Depression: The PHQ-9 scale was used, which consists of nine questions divided into four levels ranging from “not at all” to “almost every day” on a 3-point scale. After completing the retest of the PHQ-9, Cronbach’s alpha coefficient of the total score was 0.85, and the PHQ-9 had good reliability in the assessment of depression in adolescents (26).

Anxiety: The GAD-7 was mainly used. The Generalized Anxiety Scale (GAS) was developed by Spitzer et al. and consists of 7 items based on a total score. The sensitivity of the Chinese version of the GAD-7 was 86.2% with a specificity of 95.5% and a Kappa value of 0.825, suggesting that the GAD-7 has a good validity scale validity (27).

BPD: The borderline subscale of PDQ-4+ was mainly used, and “yes” and “no” were used for scoring, “yes” was scored as 1, and “no” was scored as 0. The PDQ-4+ was mainly used for scoring. “The PDQ-4+ retest reliability coefficients ranged from 0.50 to 0.80 (P < 0.01) with split-half reliability indices ranging from 0.50 to 0.93 and alpha coefficients ranging from 0.56 to 0.78; this is suitable for use in screening scales (28).

Problem behaviors: alcohol, tobacco, gambling, and the presence of addictive or psychoactive substance use were included, with presence scored as “1” and absence scored as “0.”

Critical incident: the Primary Care Post-Traumatic Stress Disorder (PC-PTSD-5) scale was used: it assesses the five symptoms of PTSD: re-experiencing, numbness, avoidance, heightened alertness, and negative change, with good diagnostic accuracy and good reliability.

School bullying: The main investigation was whether the subject had suffered from school bullying in the past 12 months. The score for having experienced bullying in school is “1”, and the score for not having experienced bullying in school is “0”.

NSSI Behavior: The main investigation is whether the subject has experienced NSSI in the past 2 weeks, with NSSI behavior being scored as “1” and no NSSI behavior being scored as “0”.

### 2.3 Statistical analysis

Descriptive analyses were performed using SPSS 26.0 software and the process of differentiating between training and validation sets, lasso regression, logistic regression, construction of column line plots, and their internal validation were performed using R software (version 4.0.0). The data were randomly divided into training and validation sets with a ratio of 7:3. The training set was used for variable selection and model construction, while the validation set was used to evaluate the effectiveness of the model. Categorical variables were described by their frequencies and percentages. Continuous variables were represented by interquartile spacing because they did not fit the normal distribution (see **Appendix A**). The training set was analyzed using univariate logistic analysis to compare the relationship of different variables with the NSSI. All tests were two-tailed and p<0.05 was statistically significant.

Significant predictors of NSSI were screened using the Lasso regression model. Lasso regression reduces the correlation between variables and ensures that subsequently generated models are not overfitted (29). 16 Logistic regression models were then constructed to further remove confounders. Combining the above methods to screen for characteristic variables, the four optimal variables were selected to construct a column-line plot. The accuracy of the model is determined by calculating the area under the curve (AUC). Calibration curves provide a visual demonstration of the consistency of the model’s predicted probabilities with actual observations. Finally, the decision curve (DCA) is used to evaluate the value of the predictive model in clinical decision-making (30).

## 3 Results

### 3.1 Incidence and demographic characteristics of NSSI in adolescents

Collinearity diagnostics revealed no substantial multicollinearity (all VIFs < 2.0; see **Appendix A**). Of the 2,081 adolescents surveyed, the age group was concentrated in the 15-18 age group, with 916 males and 1,165 females; 957 were in the first year of high school, 868 in the second year, and 256 in the third year of high school. The total number of those who had experienced NSSI was 187, with an overall detection rate of 9.0% (187/2081); the NSSI detection rate for males (4.3%) was lower than that for females (12.7%), which was statistically different. Lower grades, poorer family finances, unclear marital status of parents, presence of problematic behaviors, experiencing a major event, having depression and anxiety, and BPD were all risk factors associated with self-injury. See **Table 1** for details.

**Table 1:** Demographic information and clinical characteristics.

	NON NSSI	NSSI	p-value^2^
**Gender**			<0.001
Male	877 (95.7%)	39 (4.3%)	
Female	1017 (87.3%)	148 (12.7%)	
**Grade**			0.013
One	852 (89%)	105 (11%)	
Two	803 (92.5%)	65 (7.5%)	
Three	239 (93.4%)	17 (6.6%)	
**Only child**			0.710
No	915 (90.8%)	93 (9.2%)	
Yes	979 (91.2%)	94 (8.8%)	
**Fathers educational**			0.257
Unclear	234 (94.4%)	14 (5.6%)	
Junior high school and below	728 (90.7%)	75 (9.3%)	
High school and colleges	754 (90.3)	81 (9.7%)	
Bachelor’s degree or above	178 (91.3%)	17 (8.7%)	
**Mothers educational**			0.074
Unclear	224 (95.3%)	11 (4.7%)	
Junior high school and below	813 (90.5%)	85 (9.5%)	
High school and colleges	701 (90.0%)	78 (10.0%)	
Bachelor’s degree or above	156 (92.3%)	13 (7.7%)	
**Fathers’ profession**			0.137
Civil servant	343 (92.2%)	29 (7.8%)	
Migrant/farmer	701 (89.4%)	83 (10.6%)	
Others	850 (91.9%)	75 (8.1%)	
**Mothers’ profession**			0.506
Civil servant	319 (92.5%)	26 (7.5%)	
Migrant/farmer	728 (90.3%)	78 (9.7%)	
Others	847 (90.1%)	83 (9.9%)	
**Economic position**			0.001
Bad	119 (76.8%)	36 (23.2%)	
Ordinary	1298 (91.5%)	120 (8.5%)	
Good	397 (92.8%)	31 (7.2%)	
**Parental marital status**			0.014
Married	1570 (91.7%)	142 (8.3%)	
Divorce	296 (88.6%)	38 (11.4%)	
Unclear	28 (80.0%)	7 (20.0%)	
**Problem behavior**			<0.001
No	1229 (94.5%)	72 (5.5%)	
Yes	665 (85.3%)	115 (14.7%)	
**PTSD**			<0.001
Median (IQR)	0.00 (0.00, 1.00)	1.00 (0.00, 3.00)	
**School bullying**			0.433
No	1822 (91.1%)	178 (8.9%)	
Yes	72 (88.9%)	9 (11.1%)	
**Depression**			<0.001
Median (IQR)	3.0 (1.0, 8.0)	8.0 (5.0, 14.0)	
**Anxiety**			<0.001
Median (IQR)	2.0 (0.0, 5.0)	6.0 (3.0, 11.0)	
**BPD**			<0.001
Median (IQR)	0.00 (0.00, 2.00)	4.00 (0.00, 8.00)	

The dataset was randomly divided into training sets and validation sets in the ratio of 7:3, as detailed in **Table 2**, and there was no significant difference between the two groups (P>0.05). The training set totaled 1457 and the number of NSSIs was 136; the validation set was 624 and the number of NSSIs was 51.

**Table 2:** Comparison of characteristics between adolescents in the training and testing set1.

	Training Cohort, N = 1,457^1^	Internal Test Cohort, N = 624^1^	P
**Gender**			0.974
Male	641 (44%)	275 (44%)	
Female	816 (56%)	349 (56%)	
**Grade**			0.447
One	682 (47%)	275 (44%)	
Two	595 (41%)	273 (44%)	
Three	180 (12%)	76 (12%)	
**Only child**			0.720
No	702 (48%)	306 (49%)	
Yes	755 (52%)	318 (51%)	
**Fathers educational**			0.379
Unclear	178 (12%)	70 (11%)	
Junior high school and below	571 (39%)	232 (37%)	
High school and colleges	567 (39%)	268 (43%)	
Bachelor’s degree or above	141 (9.7%)	54 (8.7%)	
**Mothers educational**			0.805
Unclear	171 (12%)	64 (10%)	
Junior high school and below	627 (43%)	271 (43%)	
High school and colleges	542 (37%)	237 (38%)	
Bachelor’s degree or above	117 (8.0%)	52 (8.3%)	
**Fathers’ profession**			0.275
Civil servant	254 (17%)	118 (19%)	
Migrant/farmer	565 (39%)	219 (35%)	
Others	638 (44%)	287 (46%)	
**Mothers’ profession**			0.574
Civil servant	238 (16%)	107 (17%)	
Migrant/farmer	575 (39%)	231 (37%)	
Others	644 (44%)	286 (46%)	
**Economic position**			0.826
Bad	167 (11%)	68 (11%)	
Ordinary	995 (68%)	423 (68%)	
Good	295 (20%)	133 (21%)	
**Parental marital status**			0.399
Married	1,189 (82%)	523 (84%)	
Divorce	241 (17%)	93 (15%)	
Unclear	27 (1.9%)	8 (1.3%)	
**Problem behavior**			0.328
No	901 (62%)	400 (64%)	
Yes	556 (38%)	224 (36%)	
**PTSD**			0.407
Median (IQR)	0.00 (0.00, 1.00)	0.00 (0.00, 1.00)	
**School bullying**			0.502
No	1,403 (96%)	597 (96%)	
Yes	54 (3.7%)	27 (4.3%)	
**Depression**			0.821
Median (IQR)	4.0 (1.0, 8.0)	4.0 (1.0, 8.0)	
**Anxiety**			0.264
Median (IQR)	2.0 (0.0, 6.0)	2.0 (0.0, 6.0)	
**BPD**			0.078
Median (IQR)	0.00 (0.00, 3.00)	0.00 (0.00, 2.00)	

^1^n (%).

^2^Pearson’s Chi-squared test; Wilcoxon rank sum test.

### 3.2 Filter variables

Data analysis was performed on the training set, where categorical variables were analyzed using univariate analysis of variance and continuous variables were tested using non-parametric tests, and the final factors screened out as having a significant effect were gender, age, grade, family economic status, problem behavior, critical incidents, anxiety and depressive moods, and borderline tendencies (all p<0.05), as detailed in **Table 3**.

**Table 3:** Results of univariate analysis in training set2.

	NON NSSI	NSSI	p-value^2^
**Gender**			<0.001
Male	618 (95.1%)	23 (4.9%)	
Female	703 (86.2%)	113 (13.8%)	
**Grade**			0.020
One	603 (88.4%)	79 (11.6%)	
Two	550 (92.4%)	45 (7.6%)	
Three	168 (93.3%)	12 (6.7%)	
**Economic position**			0.004
Bad	140 (83.3%)	27 (16.7%)	
Ordinary	907 (91.2%)	88 (8.8%)	
Good	274 (92.9%)	21 (7.1%)	
**Problem behavior**			<0.001
No	848 (94.1%)	53 (5.9%)	
Yes	473 (85.1%)	83 (14.9%)	
**PTSD**			<0.001
Median (IQR)	0.00 (0.00, 1.00)	1.00 (0.00, 3.00)	
**Depression**			<0.001
Median (IQR)	3.0 (0.0, 7.0)	9.0 (5.0, 14.0)	
**Anxiety**			<0.001
Median (IQR)	2.0 (0.0, 5.0)	7.0 (4.0, 11.0)	
**BPD**			<0.001
Median (IQR)	0.00 (0.00, 2.00)	5.00 (0.00, 8.00)	

**Figure 1:**
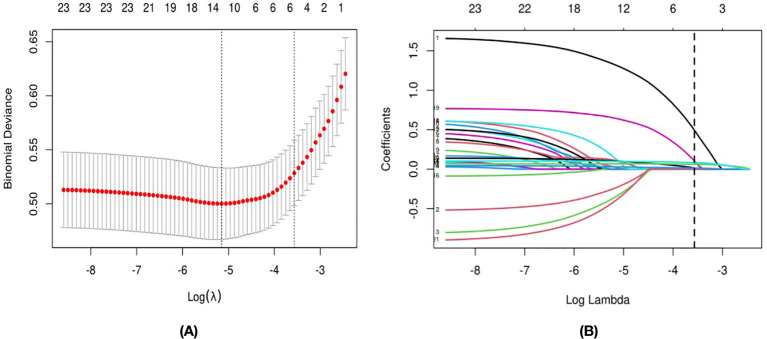
The Variable Filtering Process of the Lasso Regression: **(A)** for the Cross-Validation Graph, **(B)** for the Coefficient Graph.

To avoid overfitting, the lasso regression analysis was continued, and the results of lasso regression are shown in **Figure 1** and **Table 4**. **Figure 1A** shows the cross-validation graph, **Figure 1B** shows the coefficient graph, and **Table 4** shows the specific values of lasso regression, the value is not 0 that is, the independent variables that have a significant effect on the dependent variable. The final factors screened out as having a significant effect were gender, problem behavior, critical incident, depression and anxiety, and BPD.

**Table 4:** The coefficients of lasso regression analysis.

Coefficient	Variable
-3.29470517	(Intercept)
0.49406598	Gender_level_2
0.10328012	Problem. behavior_level_1
0.01385564	PTSD_level_
0.06230603	Depression level_
0.01326870	Anxiety level_
0.08360713	BPD_level_

To further exclude the influence of confounding factors on the results, multifactorial logistic regression was adopted, as detailed in **Table 5**. Eventually, through the above two screening methods, four NSSI-related risk factors were screened out, which were gender, problem behavior, depression score, and BPD.

**Table 5:** Multivariable logistic model of the NSSI.

Characteristic	N	Event N	OR^1^	95% CI^1^	p-value
Gender					
Male	641	23	—	—	
Female	816	113	5.07	3.09, 8.32	<0.001
Problem behavior					
No	901	53	—	—	
Yes	556	83	2.30	1.53, 3.47	<0.001
PTSD	1,457	136	1.12	0.99, 1.27	0.060
Depression	1,457	136	1.08	1.02, 1.13	0.004
Anxiety	1,457	136	1.03	0.97, 1.09	0.377
BPD	1,457	136	1.11	1.04, 1.18	0.001

^1^OR, Odds Ratio; CI, Confidence Interval.

**Table 6** shows the logistic regression model after removing all overfitting factors and confounders, showing better results (all P < 0.05).

**Table 6:** Final multivariable logistic model of the NSSI.

Characteristic	N	Event N	OR^1^	95% CI^1^	p-value
Gender					
Male	641	23	—	—	
Female	816	113	5.01	3.06, 8.20	<0.001
Problem behavior					
No	901	53	—	—	
Yes	556	83	2.37	1.58, 3.57	<0.001
Depression	1,457	136	1.10	1.06, 1.14	<0.001
BPD	1,457	136	1.13	1.07, 1.21	<0.001

^1^OR, Odds Ratio; CI, Confidence Interval.

### 3.3 Establish nomogram

**Figure 2:**
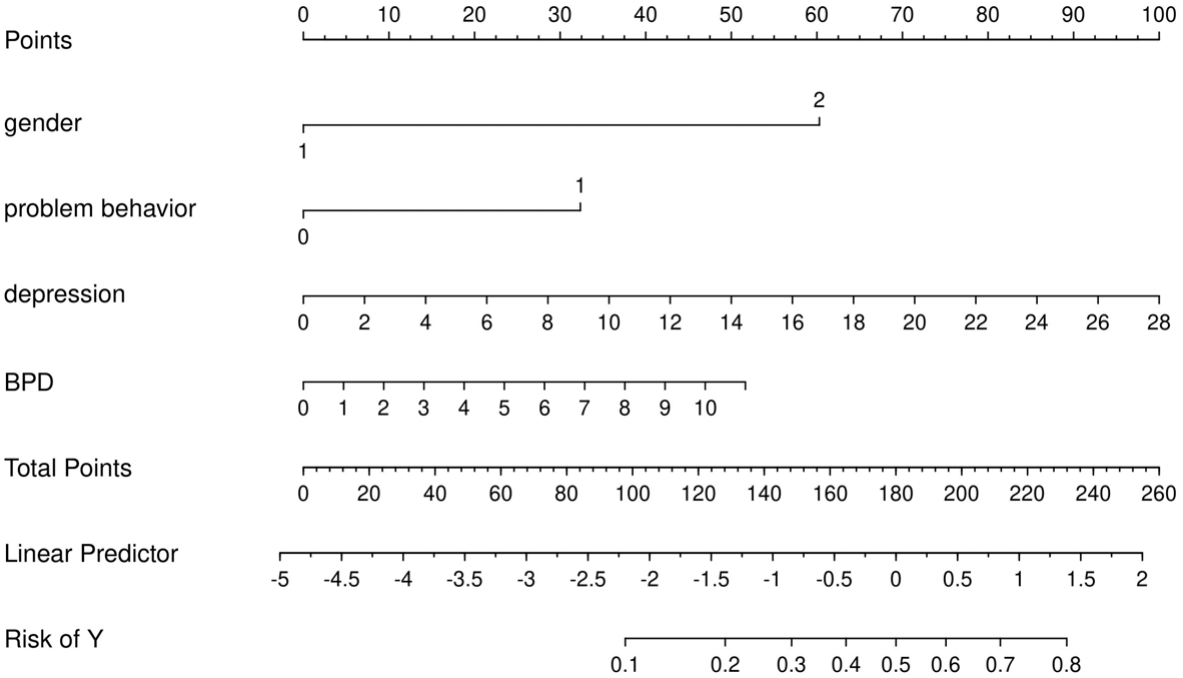
Proposed Nomogram for NSSI. (Gender: 1 = Male, 2 = Female; Problem Behavior: 0 = No, 1 = Yes; Depression: continuous score for depressive symptoms; BPD: continuous score for Borderline Personality Disorder traits).

Four high-risk factors for NSSI were selected, and a nomogramical model was developed to predict the propensity for NSSI. Each factor had a corresponding score, and these 4 scores were added up to obtain a total score, which then corresponded to the following risk scales to obtain the probability of NSSI. The higher the total score, the higher the probability of NSSI. (See **Figure 2**).

### 3.4 Internal validation

**Figure 3:**
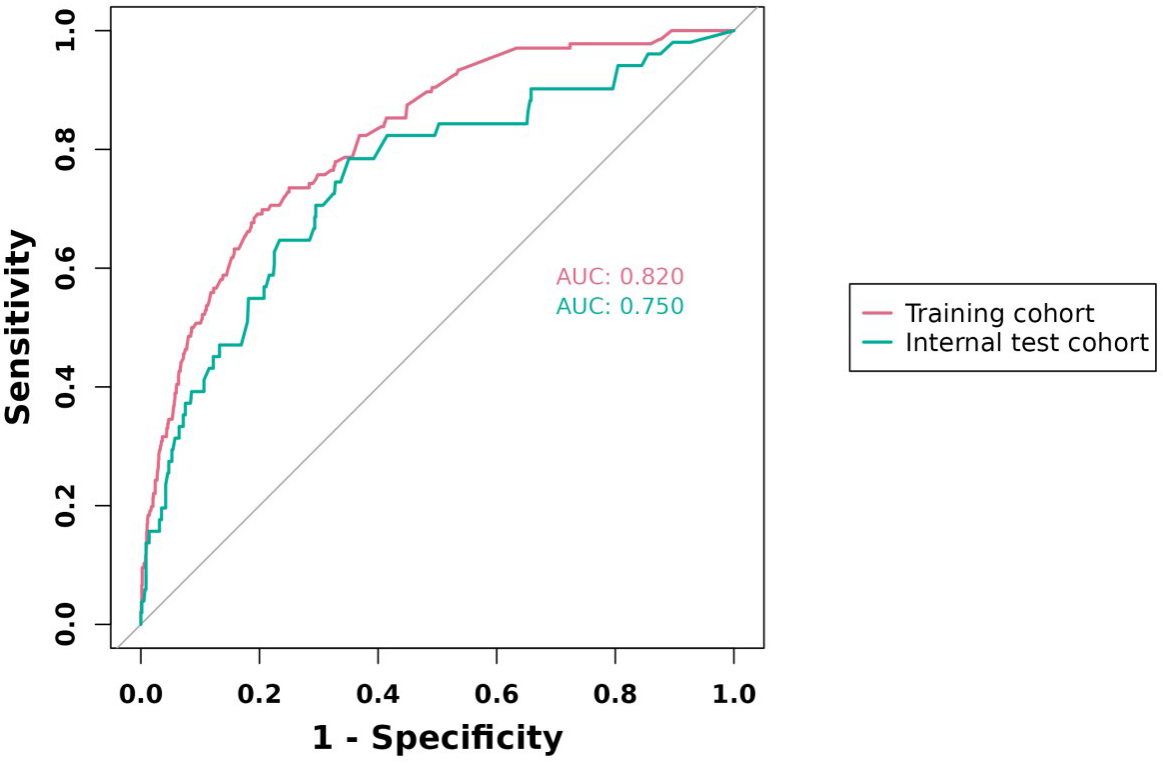
ROC curves of the study’s generated nomogram in the study.

The predictive model was evaluated using its area under the curve (AUC). The larger the value of AUC, the higher the accuracy of the model. The AUCs of the column-line plots for the training and validation sets are 0.820 and 0.750, respectively, as shown in **Figure 3**, indicating that the model is moderately accurate. The predictive ability of the model is fair.

**Figure 4:**
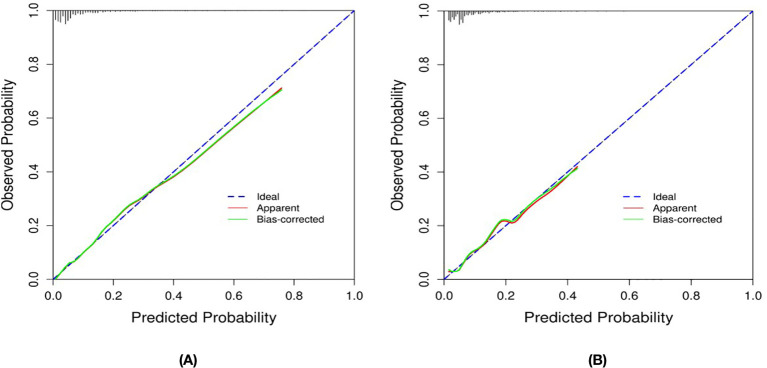
Calibration Curves of the Nomogram in the Study: **(A)** for the Training set; and **(B)** for the Internal Validation set.

The closer the Bias-corrected line or Apparent line is to the Ideal line, the higher the consistency between the predicted and actual values, using the Calibration curves to judge the model fit. As shown in **Figure 4**, both groups of Bias-corrected lines are closer to the Ideal line, which indicates a better model fit.

**Figure 5:**
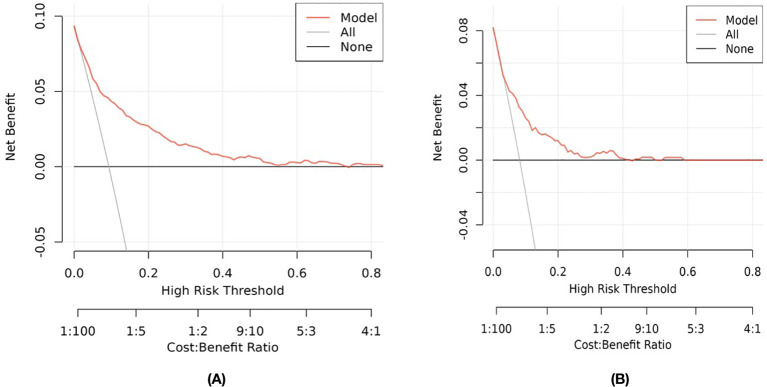
Decision Curve Analysis (DCA) for the Study’s Nomogram: **(A)** for the Training Set; and **(B)** for the Internal Validation Set.

The clinical benefits of the model were assessed using decision curve analysis (DCA). As detailed in **Figure 5**, when the model curve is above the “ALL” and “NONE” curves, the model is well-identified, calibrated, clinically applicable, and generic in this range.

## 4 Discussion

In this study, four major risk factors for NSSI were screened by Lasso regression and logistic regression analyses: females, problem behavior (addictive behaviors such as smoking, drinking, and gambling), depressed mood, and BPD, which is similar to the findings of previous studies (18, 31–37), The importance of these factors in predicting the risk of NSSI was further validated. To make the study results more intuitive and easier to understand, we visualized these risk factors by constructing a nomogram. The nomogram not only visualizes the weight and influence of each factor but also helps educators and clinicians to identify high-risk individuals more accurately.

Compared with men, women are more likely to experience NSSI (15, 38). This phenomenon may be related to physiological mechanisms, as women are affected by luteinizing hormones and estrogen, and their ability to recover from stress is slower than men’s, which increases the risk of NSSI (39, 40); In addition, females tend to ruminate more than males, and are more sensitive to changes in negative emotions, which makes it easier for them to detoxify their negative emotions or regulate their interpersonal relationships through NSSI (41, 42). Meanwhile, research findings suggest that the more severe the depressive mood, the more likely NSSI will occur (43–45), The behavior of NSSI in depressed patients may be associated with genetic and cognitive risk factors such as reduced serotonin transmission (46–48). Problem behavior may negatively impact adolescents’ physical and mental health, disrupting normal developmental trajectories and leading to an increase in mood symptoms, thereby increasing the incidence of NSSI (17, 49). Research has shown that BPD characteristics such as emotion regulation, self-punishment, and resistance to dissociation are strongly associated with NSSI behavior (50), and can indirectly increase the frequency and risk of NSSI by increasing the emotion regulation effect following NSSI (37). During the NSSI, BPD people, who are no longer sensitive to the painful experience of NSSI and whose emotional experience is no longer intense, may be at risk for NSSI because they are no longer sensitive to the painful experience of NSSI (51–54).

## 5 Strengths and limitations

First, this study avoids overfitting the model through lasso regression and logistic regression. Second, the study focuses on this special and often neglected group of students in Chinese secondary vocational schools, filling a gap in attention to their mental health. Finally, the constructed column-line diagram model visualized the risk factors of NSSI, which made the prediction model more intuitive and practical, and helped people to assess the risk of NSSI in individuals quickly.

However, the study also has shortcomings. First, the sample was limited to students of a vocational high school in Wuhan, which lacked generalization. Second, self-reports may be underreported or exaggerated, and more reliable survey methods are needed. Finally, the study was cross-sectional in design and did not focus on developmental factors, which is a limitation of the study of NSSI development.

## 6 Conclusion

This study confirms that gender, depressive symptoms, problematic behaviors, and BPD serve as core predictors of NSSI among vocational school students. The visual predictive model constructed from these factors offers practical pathways for tiered intervention in campus mental health practices. Educators can rapidly identify high-risk individuals through scale-based risk evaluation during routine academic management. Concurrently, psychological counselors may design stepwise intervention plans according to specific risk factor profiles, such as the “depression-behavioral issues” subtype or “personality trait-dominant” subtype.

Future investigations should prioritize multicenter controlled trials to assess cross-cultural applicability while strengthening comparative analyses with existing models, including traditional psychological assessment tools and emerging computational approaches. Such efforts will enhance the ecological validity and implementation precision of risk prediction systems within vocational education settings, while addressing inherent limitations of self-reported data.

## Data Availability

The raw data supporting the conclusions of this article will be made available by the authors, without undue reservation.
