# Supplementary material for: A predictive model of non-suicidal self-injury - a study based on the construction and validation of a nomogram
Source: Front Psychiatry. 2025 Apr 4;16:1539884. doi: 10.3389/fpsyt.2025.1539884 (PMC12006177; doi:10.3389/fpsyt.2025.1539884)
Supplement: Supplementary file 1 [file SupplementaryFile1.docx]

**Appendix A:**

**Data Distribution:**


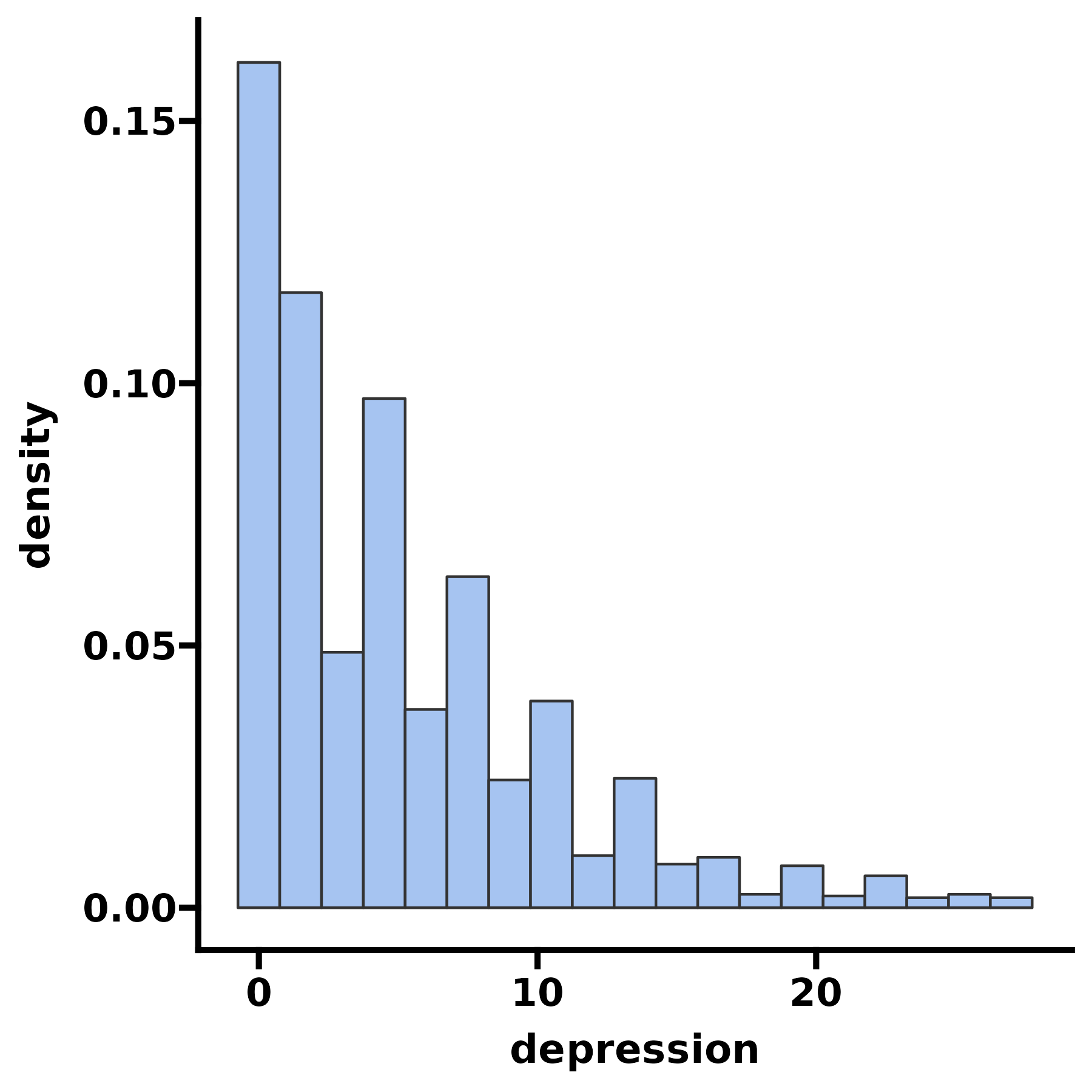


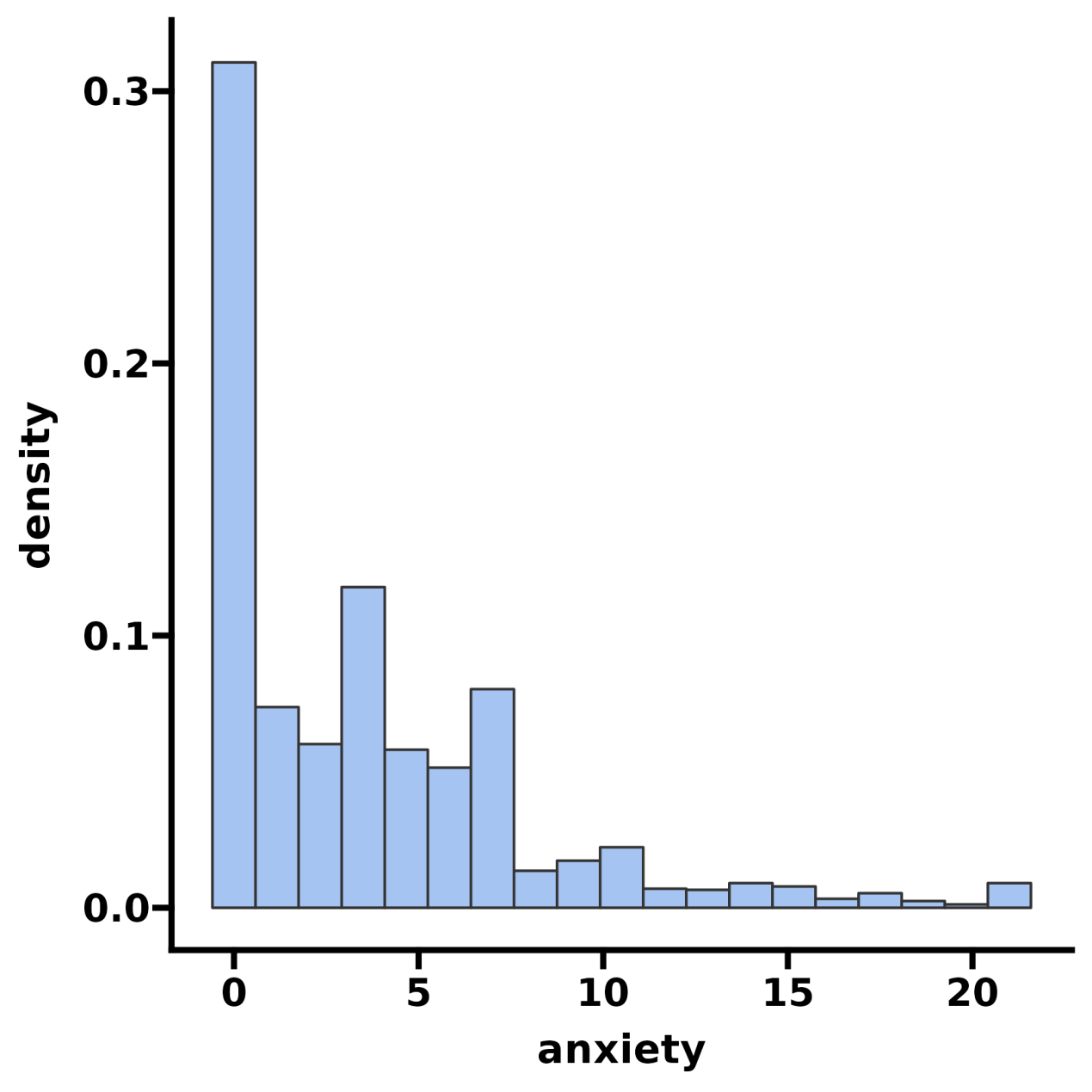


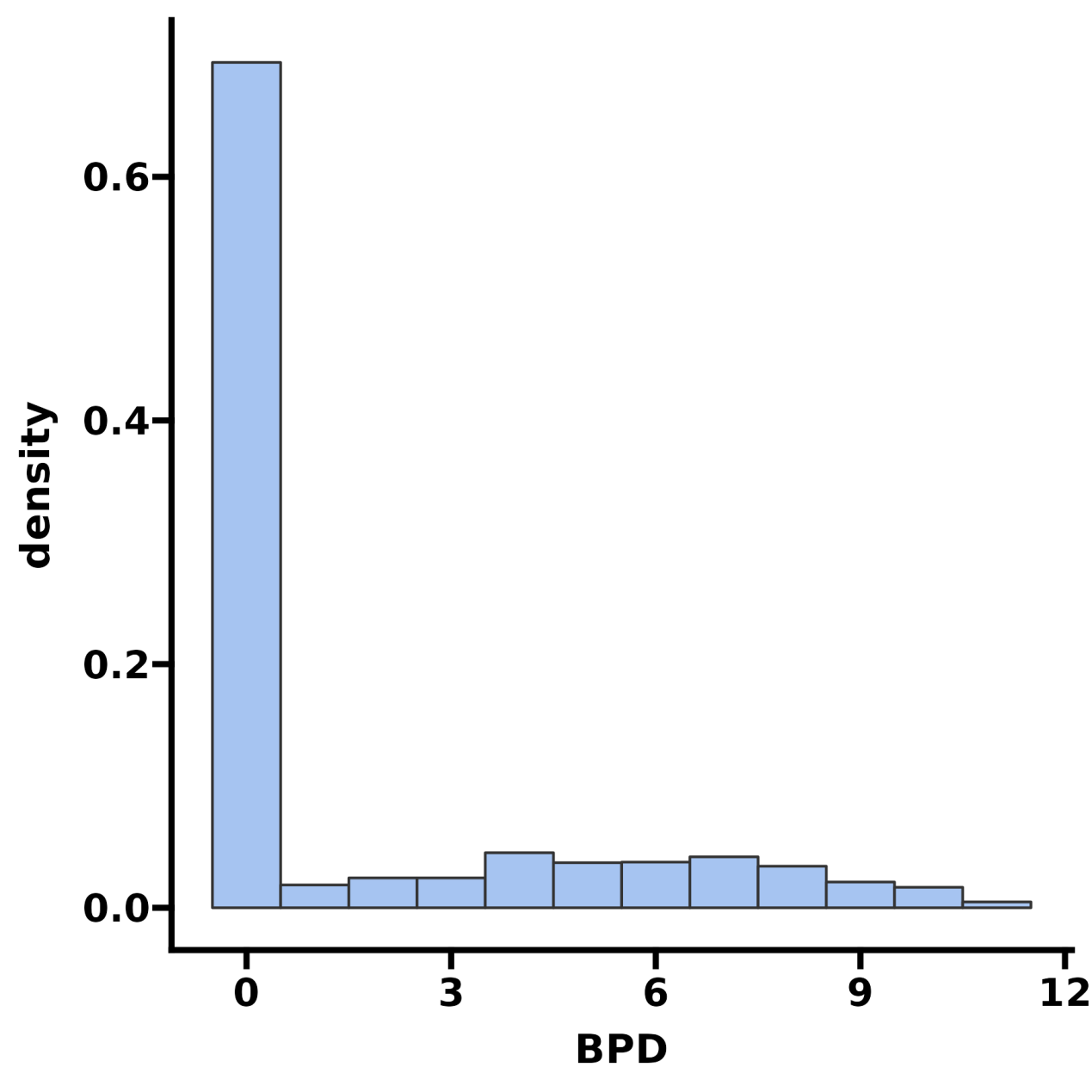


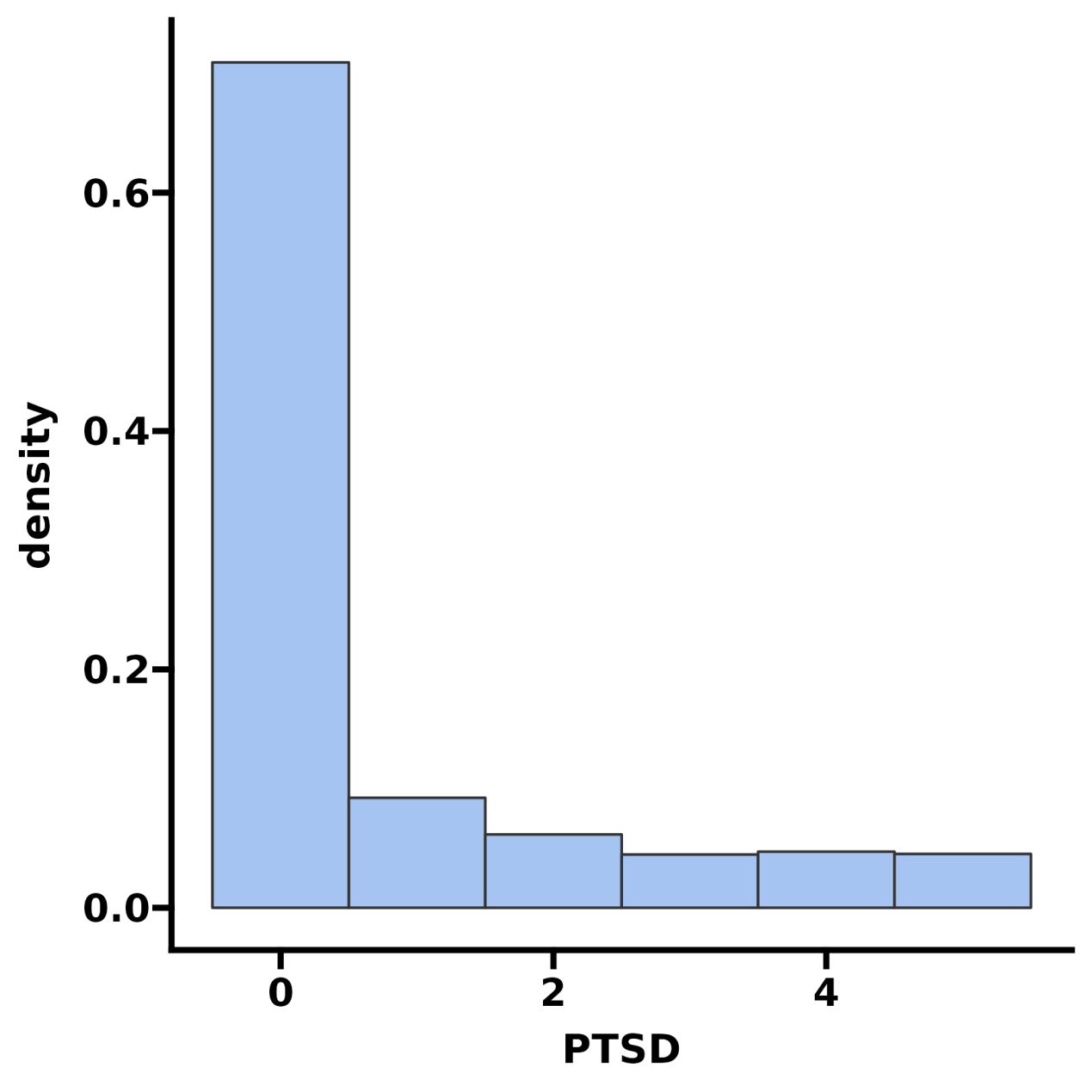


VIF

| Model | B | ​SE | Beta | t | p | TOL | VIF |
| --- | --- | --- | --- | --- | --- | --- | --- |
| (constant) | -.184 | .047 |  | -3.911 | .000 |  |  |
| gender | .082 | .012 | .142 | 6.668 | .000 | .926 | 1.080 |
| only child | -.002 | .012 | -.004 | -.184 | .854 | .885 | 1.130 |
| fathers educational | .000 | .009 | .001 | .050 | .960 | .595 | 1.682 |
| mothers educational | .013 | .010 | .035 | 1.301 | .193 | .575 | 1.739 |
| fathers’ profession | -.003 | .009 | -.009 | -.402 | .688 | .840 | 1.191 |
| mothers’ profession | .012 | .009 | .030 | 1.333 | .183 | .812 | 1.232 |
| economic position | -.003 | .011 | -.006 | -.286 | .775 | .945 | 1.058 |
| parental marital status | .016 | .014 | .024 | 1.143 | .253 | .959 | 1.042 |
| problem behavior | .065 | .013 | .110 | 5.071 | .000 | .892 | 1.121 |
| PTSD | .012 | .005 | .059 | 2.454 | .014 | .723 | 1.384 |
| school bullying | -.038 | .032 | -.026 | -1.202 | .230 | .924 | 1.082 |
| depression | .007 | .002 | .131 | 3.830 | .000 | .358 | 2.791 |
| anxiety | .003 | .002 | .051 | 1.504 | .133 | .363 | 2.751 |
| BPD | .010 | .002 | .107 | 4.226 | .000 | .653 | 1.532 |

**Data Collection Procedure​**

1.Implementation Protocol​

​Platform & Setting: The survey was administered online via Questionnaire Star (a GDPR-compliant Chinese platform) during scheduled computer lab sessions at the participating vocational school. Teachers provided technical support but had no access to response data.

​2. Administration Protocol

​Pre-survey Training: Research assistants conducted 20-minute briefings to explain item definitions (e.g., "intentional injury" vs. accidental harm).

​Time Control: Questionnaires were completed within 45 minutes under proctored conditions to minimize distractions.

​Mandatory Response: Key psychological items (e.g., PHQ-9) required answers to proceed, reducing missing data

​3. Confidentiality Safeguards

​Anonymization: Student IDs were replaced with encrypted codes (e.g., WHVHS2022-001).

​Data Encryption: All responses were AES-256 encrypted during transmission/storage

​Access Restriction: Raw data accessible only to PI and biostatisticians via two-factor authentication

​4. Informed Consent Process

Electronic consent forms were signed by both students and guardians through: Student Interface: Clicking "I agree" after reading digital instructions.

​Guardian Verification: SMS confirmation links sent to registered parent phone numbers.

​5. Post-collection Validation:

5% of participants (n=104) were randomly selected for follow-up interviews to verify response authenticity.
